# Transcriptomics resources of human tissues and organs

**DOI:** 10.15252/msb.20155865

**Published:** 2016-04-04

**Authors:** Mathias Uhlén, Björn M Hallström, Cecilia Lindskog, Adil Mardinoglu, Fredrik Pontén, Jens Nielsen

**Affiliations:** ^1^ Science for Life LaboratoryKTH ‐ Royal Institute of TechnologyStockholmSweden; ^2^ Department of ProteomicsKTH ‐ Royal Institute of TechnologyStockholmSweden; ^3^ Novo Nordisk Foundation Center for BiosustainabilityTechnical University of DenmarkHørsholmDenmark; ^4^ Department of Immunology, Genetics and PathologyScience for Life LaboratoryUppsala UniversityUppsalaSweden; ^5^ Department of Biology and Biological EngineeringChalmers University of TechnologyGothenburgSweden

**Keywords:** genome‐scale metabolic models, proteomics, transcriptomics, Genome-Scale & Integrative Biology, Metabolism, Methods & Resources

## Abstract

Quantifying the differential expression of genes in various human organs, tissues, and cell types is vital to understand human physiology and disease. Recently, several large‐scale transcriptomics studies have analyzed the expression of protein‐coding genes across tissues. These datasets provide a framework for defining the molecular constituents of the human body as well as for generating comprehensive lists of proteins expressed across tissues or in a tissue‐restricted manner. Here, we review publicly available human transcriptome resources and discuss body‐wide data from independent genome‐wide transcriptome analyses of different tissues. Gene expression measurements from these independent datasets, generated using samples from fresh frozen surgical specimens and postmortem tissues, are consistent. Overall, the different genome‐wide analyses support a distribution in which many proteins are found in all tissues and relatively few in a tissue‐restricted manner. Moreover, we discuss the applications of publicly available omics data for building genome‐scale metabolic models, used for analyzing cell and tissue functions both in physiological and in disease contexts.

## Introduction

**Figure 1:**
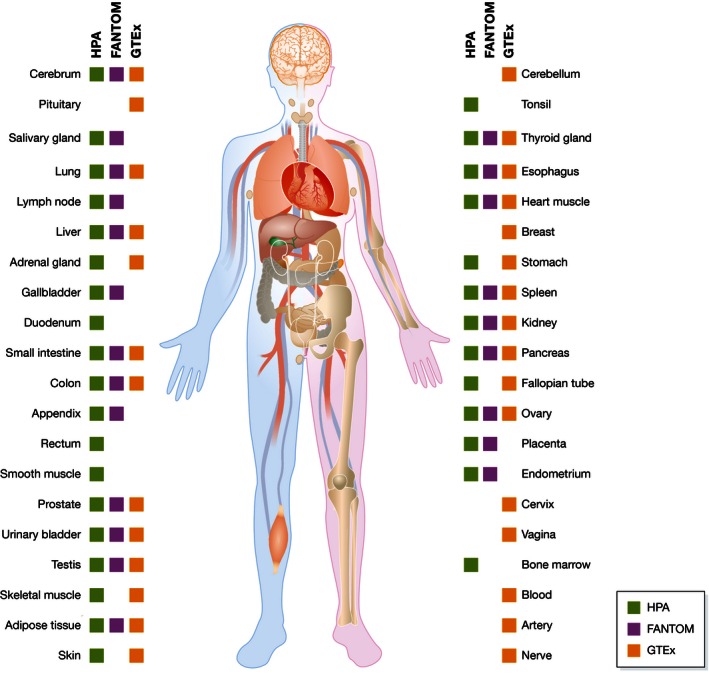
Global transcriptomics analysis of human tissues and organs Overview of the tissues and organs analyzed using RNA‐seq by the Human Protein Atlas consortium (HPA, green), tissues studied with cap analysis gene expression (CAGE) within the FANTOM consortium (purple), and tissues analyzed using RNA‐seq by the genome‐based tissue expression consortium (GTEx, orange). Altogether, 22 tissues and organs were studied with both the HPA and FANTOM datasets, while 21 tissues overlapped between the HPA and GTEx datasets.

The global classification of the human proteome with regard to its spatiotemporal expression patterns and its functions represents one of the major challenges for studying human biology and disease (Lamond *et al*, 2012). Recently, genomic, transcriptomic, and proteomic technologies have been employed to analyze the human proteome on a genome‐wide level. Genome annotation efforts, such as Ensembl (Cunningham *et al*, 2015) and Gencode consortium (Harrow *et al*, 2012) in the Encode project (Nilsson *et al*, 2015), have identified approximately 20,000 genes coding for proteins, and the UniProt consortium (UniProt, 2015) has manually annotated the majority of these genes. On the transcript level, expression levels of human genes have been monitored to study the effects of diseases, treatments, and developmental stages using microarray‐based gene expression profiling (Brawand *et al*, 2011; Petryszak *et al*, 2015). Recently, several efforts have been published with the quantitative analysis of RNA levels based on next‐generation sequencing in samples representing most of the major organs and tissues in the human body (Fig 1), including the Fantom consortium (Yu *et al*, 2015), the Human Protein Atlas (HPA) consortium (Uhlen *et al*, 2015), and the genome‐based tissue expression (GTEx) consortium (Keen & Moore, 2015). On the protein level, several large‐scale studies based on mass spectrometry analysis have also been published (Kim *et al*, 2014; Wilhelm *et al*, 2014), and these studies have been complemented with antibody‐based protein profiling using tissue microarrays containing samples representing most major tissues and organs in the human body (Fagerberg *et al*, 2014; Uhlen *et al*, 2015). Most of the quantitative data on the expression of protein‐coding genes are based on recent transcriptomics studies based on RNA‐seq. Here, we review some of the publicly available human transcriptome resources and discuss tissue data from independent research groups.

An interesting aspect of the integration of omics technologies is the sampling that depends on the sensitivity and the resolution of each technology. Consequently, the analysis of tissue samples is normally performed on a mixture of cell types using transcriptomics and mass spectrometry‐based proteomics, whereas *in situ* hybridization techniques, successfully applied for mapping the distribution of transcripts in the brain (Hawrylycz *et al*, 2012), and more qualitative approaches involving antibody‐based profiling allow analyzing single cells in their natural environment to reveal the differences in protein expression levels between neighboring cells. Thus, antibody‐based protein profiling complements quantitative transcriptomics and proteomics, based on a mixture of cell types, to reach single‐cell resolution in the analysis of gene expression in complex tissues. Recently, single‐cell technologies have been developed for transcriptomics (Tang *et al*, 2009), but no global analysis across many tissues has yet been published using such methodology. In the near future, the possibility to move to single‐cell analyses of tissues for transcriptomics and proteomics will allow analyzing cell‐to‐cell variability, which is particular interesting, that is, in the context of cancer. Moreover, the development of more quantitative assays for immunohistochemistry using fluorescent probes will be valuable for providing quantitative data for whole‐cell modeling (Stadler *et al*, 2013).

The correlation between mRNA levels and the corresponding protein levels is an important issue for the comparability of the different omics‐based technologies, and the presence or absence of such correlation on an individual gene/protein level has been debated in the literature for many years (Anderson & Seilhamer, 1997; Tian *et al*, 2004; Gry *et al*, 2009; Maier *et al*, 2009, 2011; Lundberg *et al*, 2010; Schwanhausser *et al*, 2011). However, a comparison of steady‐state levels of mRNA and proteins in human cell lines using RNA‐seq and quantitative triple‐SILAC analysis showed good genome‐wide correlations when the mRNA and protein levels of an individual gene were compared in three separate cell lines (Lundberg *et al*, 2010). These observations were supported by Kuster and coworkers (Wilhelm *et al*, 2014) comparing mass spectrometry data from different tissues with RNA‐seq data obtained from the HPA consortium (Uhlen *et al*, 2015). Again, the steady‐state levels for individual genes correlated across several tissues. Overall, these studies suggest that the amount of a given protein in a cell or tissue is, in general, reflected by the corresponding mRNA level, although this gene‐/protein‐specific ratio may vary greatly between different gene products depending on various factors, mainly translational rates and protein half‐lives (Eden *et al*, 2011), and the transcript level for a given gene might therefore be used to predict the corresponding protein level. This hypothesis needs to be confirmed by more in‐depth studies using quantitative measurements at both the RNA and protein levels. However, it suggests that next‐generation sequencing of the transcriptome is a powerful tool for indirect measurements of protein expression levels, thus forming an attractive link between the field of genomics and proteomics.

## Analyses of the human transcriptome by different methods

Several genome‐wide transcriptome profiling methods have been used for identifying and quantifying global gene expression patterns, each allowing for a quantitative analysis of RNA transcripts. Whole‐body maps based on microarray analyses have been launched, such as BioGPS (Wu *et al*, 2009), and these have recently been followed by several tissue‐based data portals based on RNA‐seq. Some of these publicly available repositories for transcriptomics data are summarized in Table 1 with a focus on datasets from RNA‐seq experiments. The resources include repositories for external data, such as Expression Atlas from the European Bioinformatics Institute (EBI) and Gene Expression Omnibus from the National Center for Biotechnology Information (NCBI), as well as repositories with internally generated transcriptome data, such as the GTEx, the Human Protein Atlas, and the Allen Brain Atlas. In a recent study (Danielsson *et al*, 2015), the concordance of RNA‐seq data from four large‐scale efforts was compared based on gene expression measurements for ostensibly similar samples (specifically, human brain, heart, and kidney samples). The authors concluded that human tissue RNA‐seq expression measurements appear quite consistent, considering that samples cluster by tissue rather than laboratory of origin given simple preprocessing transformations.

**Table 1:** Data resources for RNA expression data with relevance for human protein‐encoding genes

Resource	Affiliation	Description	Link (URL)	References
Human Protein Atlas	Science for Life Lab (Sweden)	Tissue‐based RNA data based on surgically removed tissues (RNA‐Seq)	http://www.proteinatlas.org/	(Uhlen *et al*, 2015)
GTEx	Broad Institute (USA)	Tissue‐based RNA data based on postmortem samples (RNA‐Seq)	http://gtexportal.org/home/	(Keen & Moore, 2015)
FANTOM	Riken Institute (Japan)	Tissue‐based RNA data based on CAGE	http://fantom.gsc.riken.jp/	(Yu *et al*, 2015)
RNA‐Seq Atlas	J. Gutenberg University (Germany)	A reference database for gene expression profiling in normal tissue by next‐generation sequencing	http://medicalgenomics.org/rna_seq_atlas	(Krupp *et al*, 2012)
Allen Brain Atlas	Allen Institute (USA)	An anatomically comprehensive atlas of the adult human brain transcriptome	http://human.brain-map.org/	(Hawrylycz *et al*, 2012)
Evolution of gene expression	University of Lausanne (Switzerland)	The evolution of gene expression levels in mammalian organs	http://www.ncbi.nlm.nih.gov/geo/query/acc.cgi?acc=GSE30352	(Brawand *et al*, 2011)
AltIso	MIT (USA)	Alternative isoform regulation in human tissue transcriptomes.	http://www.ncbi.nlm.nih.gov/geo/query/acc.cgi?acc=GSE12946	(Wang *et al*, 2008)
Expression Atlas	EBI (UK)	Repository for RNA expression data (both microarray and RNA‐Seq)	https://www.ebi.ac.uk/gxa	(Petryszak *et al*, 2015)
ArrayExpress	EBI (UK)	International functional genomics public data repositories	http://www.ebi.ac.uk/arrayexpress/	(Rustici *et al*, 2013)
Illumina Body Map	Illumina (USA)	RNA‐Seq of 16 human individual tissues	http://www.ebi.ac.uk/arrayexpress/experiments/E-MTAB-513/	(Rustici *et al*, 2013)
Gene Expression Omnibus	NCBI (USA)	Repository for RNA expression data (both microarray and RNA‐Seq)	http://www.ncbi.nlm.nih.gov/geo/	(Barrett *et al*, 2013)

An alternative approach to RNA‐seq, named cap analysis gene expression (CAGE), has been described by the Fantom consortia (Yu *et al*, 2015) and allows for quantitative measurements of transcripts based on sequencing the 5′‐end of capped mRNA molecules. The correlation between RNA‐seq and CAGE for transcriptome analysis was recently investigated (Yu *et al*, 2015), and the transcriptome of 22 tissues was analyzed using both methods (Fig 1) based on 79 RNA‐seq (HPA) and 27 CAGE (FANTOM) samples. Tissue‐to‐tissue comparisons showed a high genome‐wide correlation between the two datasets (Yu *et al*, 2015). Interestingly, discrepancies between the two datasets can largely be explained by gene model annotation issues or technical artifacts inherent in the respective methodologies. As an example, the HPA data excluded mRNA without poly‐adenylation tails and it is therefore not surprising that many histone genes were lacking in the RNA‐seq data, but are present in the CAGE data. Conversely, CAGE peaks mapping more than 500 base pairs from the transcriptional start site are lacking in the CAGE dataset, as well as CAGE peaks mapping to two or more locations on the genome, which are removed from the dataset. Thus, the two methods are complementary and it would be attractive to integrate data obtained by these two approaches to refine gene models and to improve the interpretation of gene expression values.

## Classification of all human protein‐coding genes based on tissue profiling

The different omics‐based analyses of the human proteome have allowed the classification of protein‐coding genes with regard to tissue‐restricted expression. In the analysis performed by the HPA consortium (Fagerberg *et al*, 2014; Uhlen *et al*, 2015), a cutoff of 1 FPKM (Hebenstreit *et al*, 2011) was used to indicate the presence or absence of transcripts for a particular gene in a tissue. Based on this definition, all human protein‐coding genes were classified into (i) genes with an elevated expression in one or several tissues, (ii) genes expressed in all analyzed tissues, (iii) genes with mixed expression found in several, but not all tissues, and (iv) genes not detected in any tissues. The elevated genes were further stratified into “tissue enriched”, “group enriched”, or “tissue enhanced”. The term “tissue specific” was avoided as it depends on the definition of cutoff values, and only few genes, including well‐known proteins such as insulin, PSA, and troponin, were found to be exclusively expressed in a single tissue type (Uhlen *et al*, 2015). A classification of all protein‐coding genes is shown in Table 2 (cutoff of 0.5 FPKM).

**Table 2:** Classification of all human protein‐coding genes based on transcript expression levels in tissues and organs. The columns HPA and GTEx indicate the number of genes identified in the different categories using the datasets (Keen & Moore, 2015; Uhlen *et al*, 2015) from these two consortia

Category	Definition	HPA	GTEx
Tissue enriched	At least fivefold higher mRNA levels (FPKM) in a particular tissue as compared to all other tissues	2,359	2,289
Group enriched	At least fivefold higher mRNA levels in a group of tissues (2–7)	1,208	1,307
Enhanced	At least fivefold higher mRNA levels in a particular tissue as compared to the average levels in all tissues	3,227	3,077
Expressed in all	Detected in all tissues	8,385	8,459
Mixed	Detected in at least two tissues, but not in all, and not part of any of the categories above	2,484	2,537
Not detected	Not present in any of the analyzed tissues (under cutoff)	1,021	1,015
Total	Total number of genes analyzed	18,684	18,684
Total elevated	Total number of tissue‐enriched, group‐enriched, and tissue‐enhanced genes	6,794	6,673

**Figure 2:**
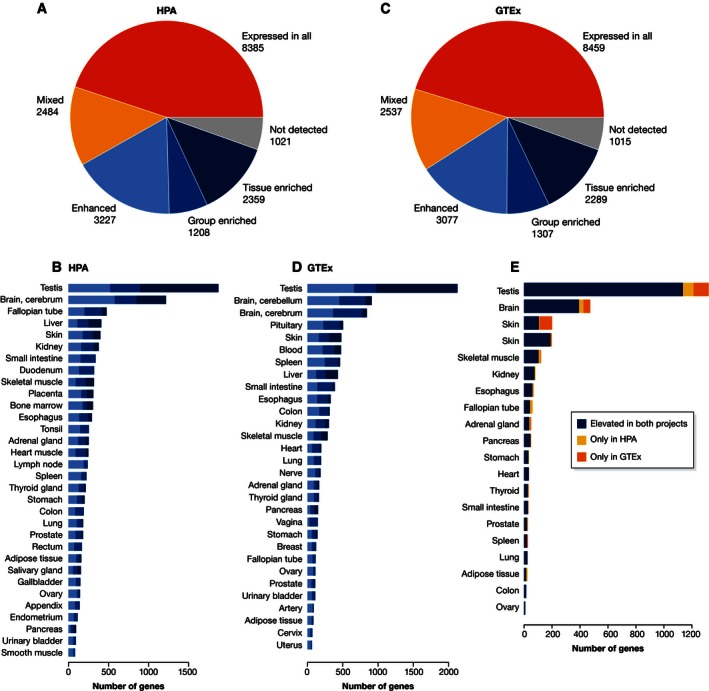
Classification of all protein‐coding genes using transcriptomics data (A) Pie chart showing the number of genes that fall into each expression specificity category, based on the classifications of HPA (32 tissues, 137 samples) (with a cutoff of 0.5 FPKM). (B) The number of protein‐coding genes classified as tissue enriched (dark blue), group enriched (medium blue), and tissue enhanced (light blue) based on the HPA dataset. (C) Pie chart showing the number of genes that fall into each expression specificity category, based on the classifications of GTEx (30 tissues, 2,510 samples) (Keen & Moore, 2015) (with a cutoff of 0.5 FPKM). (D) The number of protein‐coding genes classified as above based on GTEx dataset. (E). Barplot showing the overlap of tissue‐enriched genes between the two datasets. All genes that are tissue enriched in either dataset are depicted. Genes classified as tissue enriched/group enriched/tissue enhanced in the same tissue in both datasets are shown in blue; genes only enriched in one of the datasets are shown in yellow (only HPA) or orange (only GTEx).

The classification of the human protein‐coding transcriptome showed that almost half of the genes were detected in all tissues (45%), while 13% showed a mixed expression (Fig 2A). Approximately one‐third of the genes showed a tissue elevated expression with 13% of the genes enriched in one of the analyzed tissues. Only 5% of the genes were not detected in any of the analyzed tissues. A further analysis of the number of genes with a tissue elevated expression (Fig 2B) showed that the testis has by far the highest number of tissue‐enriched genes followed by the brain (cerebral cortex) and liver.

The recently published RNA‐Seq data generated by the GTEx consortium (Bahcall, 2015; GTEx Consortium, 2015; Gibson, 2015) allow for an independent tissue‐based classification of the human proteome. The GTEx dataset includes more than 1,600 postmortem samples from mostly overlapping, but in some cases unique, tissues compared to the HPA consortium (Fig 1). For example, the GTEx dataset includes more tissue samples from the brain, blood, and nerves, which are not included in the HPA dataset. As illustrated in Fig 2C, the overall tissue‐based classification based on the GTEx dataset and an identical cutoff of 0.5 FPKM is similar to that of the HPA with 45% of the genes expressed in all tissues, 14% showing a mixed expression, 12% being tissue‐enriched expression, and 5% of the genes not detected in any of the analyzed tissues. With respect to tissue‐elevated genes (Fig 2B and D) the testis is again observed to contain the largest number of tissue‐enriched genes, followed by the brain (cerebellum, cortex, and pituitary), skin, and liver. The values for the HPA and the GTEx datasets can be found in Table EV1.

## Tissue‐enriched genes vs. ubiquitously expressed genes

**Figure 3:**
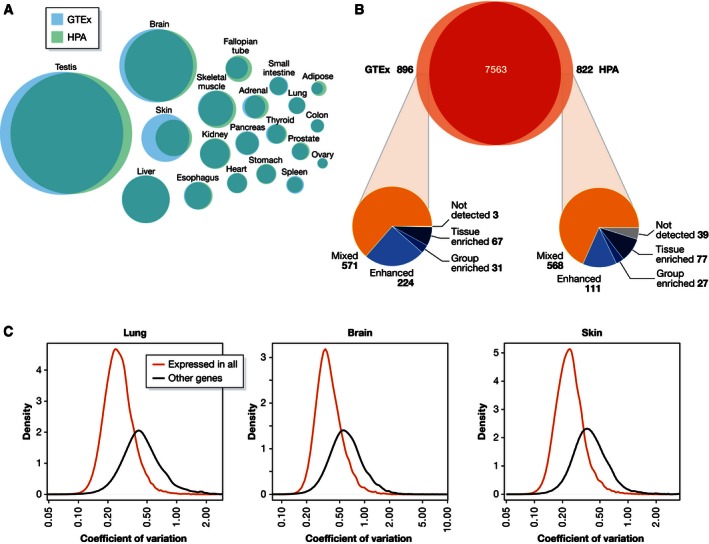
Protein classification and interindividual variations (A) Venn diagrams showing the overlap between tissue‐elevated genes between the two datasets, HPA in light green and GTEx in light blue. (B) Venn diagram showing the overlap between genes classified as “expressed in all tissues” between the two datasets. The pie charts show the classification of the non‐overlapping genes in the dataset where the gene was not detected in all tissues. (C) Comparison of interindividual variation between genes that are annotated as “expressed in all tissues” and all other genes, in lung, brain, and skin (these tissues were selected because they have a large number of biological replicates). The plots illustrate the distribution of the coefficient of variation (CV) within the tissue for all genes in each of the two classes (red: expressed in all, black: other). The CV is shifted toward the lower side in the “expressed in all” category (*P *≪ 0.001), suggesting that genes that are expressed in all tissues have lower variation between individuals.

Tissue‐enriched genes identified by the analysis of the HPA data (Uhlen *et al*, 2015) based on the definitions shown in Table 2 can be found for all tissues in the interactive HPA database (www.proteinatlas.org/humanproteome/tissue+specific). A functional Gene Ontology analysis of the tissue‐enriched genes in the HPA dataset has been performed and the results are consistent with the function of each tissue (Uhlen *et al*, 2015). As an example, genes elevated in liver encode secreted plasma and bile proteins, detoxification proteins, and proteins associated with metabolic processes and glycogen storage (Kampf *et al*, 2014), whereas genes elevated in adipose tissue encode proteins involved in lipid metabolic processes (Mardinoglu *et al*, 2014b) and genes elevated in skin encode proteins associated with functions related to the barrier function (squamous cell differentiation and cornification), skin pigmentation, and hair development (Edqvist *et al*, 2015). In order to further validate these lists, we have compared the overlap of tissue‐enriched genes identified using the independent HPA and GTEx datasets. The number of tissue‐enriched genes in the different tissues and the overlap between the two datasets are shown in Figs 2E and 3A, and Table EV2. Overall, it is reassuring that there is a significant overlap in the tissue classification of the genes based on the two independent datasets. The fact that similar results are obtained when using fresh frozen tissue (HPA) and postmortem tissue (GTEx) suggests negligible effects of the sampling procedures used by the GTEx consortium on RNA degradation. In the comparison, note that in the HPA dataset, the brain contains only one tissue (cerebral cortex), while the corresponding GTEx dataset is based on three different tissues (cerebellum, cortex, and pituitary). The large discrepancy for skin can be explained by the fact that the sampling of skin in the HPA was based on shave biopsies including mainly epidermis (Uhlen *et al*, 2015), while the GTEx consortium also included the underlying dermis, most likely containing skin adnexal structures such as hair follicles and sweat glands.

Genes identified as “expressed in all tissues” are expected to be either “housekeeping” genes for which the protein product is needed in every cell, such as proteins involved in transcription, translation, and energy metabolism, or genes expressed in cell types that are present in all tissue types, such as lymphocytes, macrophages, fibroblasts, and endothelial cells. HPA and GTEx data largely overlap for this category as well, with 7,563 genes identified as “expressed in all” by both resources (Fig 3B). Between 800 and 900 genes were only identified in one of the two datasets, and a more detailed analysis shows that the vast majority of these genes were identified as “mixed” by the other dataset (Fig 3B). This suggests that these genes move between categories based on the relatively arbitrary FPKM cutoff and indicates that expression in a single tissue below the detection threshold makes a gene move from “expressed in all” to “mixed”.

## The variation in protein profiles between individuals

A relevant question arising is the level of interindividual variation in gene expression levels. A comparison of individual variation for “housekeeping” genes (defined as expressed in all analyzed tissues) and genes with a more tissue‐restricted expression using GTEx data is presented in Fig 3C for three different tissues (lung, brain, and skin) that are represented by a large number of biological replicates. For all three tissues, the coefficient of variation (CV) in the “expressed in all” category shifted toward the lower side, suggesting that genes expressed in all tissues seem to vary less between individuals for a particular tissue as compared to genes with a tissue‐restricted expression pattern. This illustrates that the proteins found in all tissues are expressed at relatively similar levels across the analyzed tissues, suggesting, as perhaps expected, that these proteins that are involved in “basic functions” are required at similar concentrations in the various tissue types.

## Building genome‐scale metabolic models for human tissues

**Figure 4:**
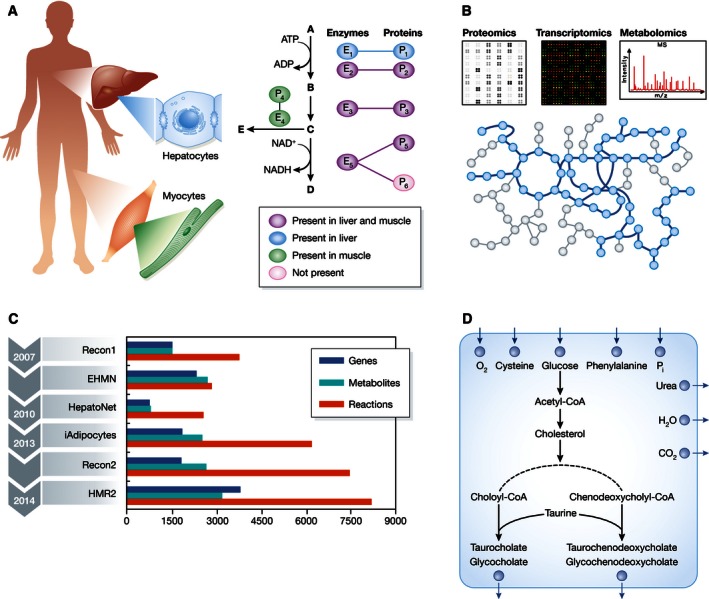
Genome‐scale metabolic models for human cells/tissues (A) GEMs incorporate the known biochemical reactions and their catalyzing enzymes in a particular cell/tissue type. The information related to the reaction–gene association is used for the reconstruction of context‐specific GEMs. (B) The continuously increasing number of reactions, metabolites, and genes included in generic human GEMs and manually curated cell‐/tissue‐specific GEMs generated in the recent years is shown. (C) High‐throughput omics data including proteomics, transcriptomics, and metabolomics have been used for reconstructing cell‐/tissue‐specific GEMs based on generic human GEMs. (D) The metabolic tasks that are known to occur in a given human cell/tissue need to be defined to generate functional cell‐/tissue‐specific GEMs. The definition of the metabolic task related to bile acid synthesis in the liver is presented. Glucose, cysteine, phenylalanine, oxygen (O_2_), and phosphate (Pi) must be taken up, whereas urea, water (H_2_O), and carbon dioxide (CO_2_) must be secreted in order to successfully simulate bile acid synthesis in liver GEM.

High‐quality genome‐wide proteomics and transcriptomics data can be used for generating and improving context‐specific biological networks including protein–protein interaction (PPI), regulatory, signaling, and metabolic networks (Papin *et al*, 2005; Qian *et al*, 2005; Bossi & Lehner, 2009) in order to gain further insights into the differences in cellular functions across tissues. Genome‐scale metabolic models (GEMs) that can be reconstructed directly from proteomics or transcriptomics data are particularly well suited for the analysis of biological functions, since they can be applied to examine the metabolic functions associated with a given cell type. Several studies have recently reported the use of proteomics data to reconstruct GEMs for analyzing metabolic processes across different cell and tissue types in humans (Mardinoglu & Nielsen, 2015; O'Brien *et al*, 2015; Yizhak *et al*, 2015; Bjornson *et al*, 2016) and mice (Mardinoglu *et al*, 2015b). GEMs contain thousands of biochemical reactions and their catalyzing protein‐coding genes in a cell/tissue, which generate a complex network of molecular interactions capturing the metabolic functions of this cell/tissue (Fig 4A). This reaction network is converted into a computational model using a stoichiometric (S) matrix and can be applied for the analysis of physiological data collected from both healthy and diseased states (Mardinoglu & Nielsen, 2012; Mardinoglu *et al*, 2013b).

The first human GEMs, Recon1 (Duarte *et al*, 2007) and EHMN (Ma *et al*, 2007), were developed nearly 10 years ago and have now grown to the level where they can be used for predicting the metabolic response of cell/tissue to a given perturbation. These integrative models allowed the identification of new drug target candidates by theoretical analyses (Frezza *et al*, 2011), and many enzymes have already been proposed as drug targets for cancer treatment (Yizhak *et al*, 2015). Recently, more comprehensive generic human GEMs including Recon2 (Thiele *et al*, 2013) and HMR2 (Mardinoglu *et al*, 2014a) were constructed by integrating the components of the first generic human GEMs with manually reconstructed context‐specific GEMs. Recon2 covers the content of the HepatoNet, a manually reconstructed GEM for hepatocytes (Gille *et al*, 2010), whereas HMR2 covers the content of both HepatoNet and *iAdipocytes1809*, a manually reconstructed GEM for adipocytes (Mardinoglu *et al*, 2013a). HMR2 also includes the extensive description of lipid metabolism present in human adipocytes and hepatocytes. The number of reactions, metabolites, and genes incorporated in each model as well as the conceptual evolution of global reconstructions of human metabolism is presented in Fig 4B. As illustrated in Fig 4B, HMR2 is the most comprehensive global reconstruction of human metabolism and this model together with other generic models of human metabolism has served as a basis for the reconstruction of context‐specific GEMs (Fig 4C).

Context‐specific GEMs were generated by manually curating the existing literature as well as by using various algorithms that have been reviewed elsewhere (Machado & Herrgard, 2014). For instance, the recently developed tINIT algorithm enables the reconstruction of simulation‐ready GEMs based on proteomics data and metabolic functions that are known to occur in the cell/tissue of interest (Agren *et al*, 2014). The implementation of a metabolic function related to bile acid synthesis into the liver‐specific GEM is shown as an example in Fig 4D. Recently, 32 tissue‐specific GEMs for healthy human tissues were generated by integrating RNA‐seq data from the HPA in combination with the tINIT algorithm and they were used to compare the metabolic differences between these tissues (Uhlen *et al*, 2015). GEMs reconstructed based on RNA‐seq data successfully predicted tissue‐specific functions. For instance, the liver GEM was the only model that could successfully perform metabolic functions related to bile acid synthesis. Moreover, the liver GEM was able to perform all defined human metabolic functions and it was the largest GEM in terms of incorporated reactions, metabolites, and genes, reflecting its high metabolic activity compared to the other analyzed tissues. A list of the various cell‐/tissue‐specific GEMs that have been generated so far, either in physiological or in disease states, is presented in Table 3.

**Table 3:** List of generic and cell‐/tissue‐specific human GEMs

Model name	Application	References
Generic human GEMs		
Recon1	Integration of genomic and bibliomic data	(Duarte *et al*, 2007)
EHMN	Integration of genomic and bibliomic data	(Ma *et al*, 2007)
HMR	Integration of previous generic human GEMs and publicly available databases	(Agren *et al*, 2012)
Recon2	Community‐based reconstruction of human metabolism	(Thiele *et al*, 2013)
HMR2	Incorporation of extensive lipid metabolism into the generic human GEM	(Mardinoglu *et al*, 2014a)
Cell‐/tissue‐specific GEMs		
Red blood cell	Analysis of the metabolic loads in red blood cells	(Wiback & Palsson, 2002)
Mitochondria	Study of the human mitochondrial metabolism	(Vo *et al*, 2004)
Fibroblasts	Metabolic alterations in Leigh syndrome	(Vo *et al*, 2007)
HepatoNet1	Investigation of hepatic enzyme deficiencies	(Gille *et al*, 2010)
Computational liver model	Discovery of biomarkers of liver disorders including hyperammonemia and hyperglutaminemia	(Jerby *et al*, 2010)
Kidney	Prediction of causal drug off‐targets that impact kidney function	(Chang *et al*, 2010)
Brain (three neuron types and astrocytes)	Revealing the metabolic alterations in Alzheimer's disease	(Lewis *et al*, 2010)
IAB‐AMQ‐1410	Analysis of the host–pathogen interactions with *Mycobacterium tuberculosis*	(Bordbar *et al*, 2010)
Multitissue (hepatocytes, myocytes, and adipocytes)	Revealing the metabolic alterations in T2D	(Bordbar *et al*, 2011a)
Erythrocyte (iAB‐RBC‐283)	Revealing the complexity in the functional capabilities of human erythrocyte metabolism	(Bordbar *et al*, 2011b)
69 cell‐specific GEMs	Studying the metabolic differences between healthy cells and cancers	(Agren *et al*, 2012)
126 tissue‐specific GEMs	Comparative analysis between healthy tissues and tumor	(Wang *et al*, 2012)
CardioNet	The effect of oxygen and substrate supply on the efficiency of selected metabolic functions of cardiomyocytes	(Karlstaedt *et al*, 2012)
*iAdipocytes1809*	Revealing the metabolic differences in obese subjects	(Mardinoglu *et al*, 2013a)
Tissue‐specific GEMs	Studying the metabolic differences between healthy tissues and cancers	(Nam *et al*, 2014)
Liver GEM	Studying urea metabolism in liver tissue	(Vlassis *et al*, 2014)
83 cell‐specific GEMs	Defining the major metabolic functions in human cell types	(Agren *et al*, 2014)
*iHepatocytes2322*	Revealing the metabolic alterations in response to NAFLD	(Mardinoglu *et al*, 2014a)
*iMyocyte2419*	Revealing the metabolic alterations in response to T2D	(Varemo *et al*, 2015)
32 tissue‐specific GEMs	Global analysis of the metabolic functions in major human tissues	(Uhlen *et al*, 2015)

## Applying context‐dependent GEMs for analyzing human diseases

Context‐specific GEMs in combination with omics data obtained in disease‐specific contexts have been used to elucidate the metabolic capabilities of cells/tissues involved in metabolism‐related disorders including obesity (Mardinoglu *et al*, 2013a, 2014b, 2015a), non‐alcoholic fatty liver disease (NAFLD) (Mardinoglu *et al*, 2014a; Hyötyläinen *et al*, 2016), type 2 diabetes (T2D) (Varemo *et al*, 2015), and aging (Yizhak *et al*, 2013), as well as to determine unique metabolic properties of cancer cells (Agren *et al*, 2012; Gatto *et al*, 2014; Nam *et al*, 2014) and even individual cell lines (Yizhak *et al*, 2014a,b; Gatto *et al*, 2015; Ghaffari *et al*, 2015) and tumors (Agren *et al*, 2014). Each of these studies advanced our understanding of the molecular mechanisms underlying these diseases and allowed the discovery of drug targets or biomarkers that can be used for designing effective treatment strategies.

Recently, a GEM for skeletal myocytes was reconstructed using cell type‐specific RNA‐seq data and incorporating cell type‐specific proteomics data from the HPA. First, the presence/absence of each enzyme in myocytes was determined and based on this information the corresponding metabolic reaction was incorporated into the myocyte‐specific GEM (Varemo *et al*, 2015). The model was employed for characterizing the metabolic alterations in skeletal muscle in response to T2D based on the meta‐analysis of six published datasets on T2D muscle gene expression. The metabolic alterations observed in the skeletal muscle T2D patients involved differences in pyruvate oxidation, tetrahydrofolate metabolism, and branched‐chain amino acid catabolism.

The interplay between a large number of biological pathways and the significant variation between patients makes it extremely difficult to identify effective drug targets and biomarkers for metabolic diseases. Personalized GEMs that account for interindividual differences as well as for the unique characteristics of disease progression in each individual (Agren *et al*, 2014) present a potential solution to these issues. In a recent study, personalized cancer GEMs for six hepatocellular carcinoma (HCC) patients as well as 83 healthy cell‐specific GEMs were reconstructed using the tINIT algorithm to integrate proteomics data from the HPA and metabolic functions that are present in human cells (Agren *et al*, 2014). Based on these personalized GEMs, anticancer drug targets that can be used for inhibiting the HCC tumor growth in each patient were identified. One of the targets, predicted to be effective in all patients, was experimentally validated in human HCC cancer cell lines. Overall, the observation that fat oxidation was increased in the analyzed HCC tumors indicated that targeting this metabolic process could be used for developing treatment strategies for HCC.

Another recent application of GEMs in the context of HCC is presented in the study of Björnson *et al* (2015). In this case, gene expression data from approximately 360 HCC tumors and 50 non‐cancerous liver samples were analyzed using a HCC‐specific GEM. Interestingly, a group of patients showed an increased fat oxidation, whereas another group showed a decreased fat oxidation. The fact that HCC tumors from different patients may have completely opposite metabolic programming highlights that careful stratification of HCC patients and personalized medicine approaches are highly advantageous for developing effective treatment strategies. Overall, these studies provide valuable insights into inter‐ and intratumor heterogeneity and point out that it might be extremely difficult to treat all different HCC patients with a single drug. This drug can be effective in the right context, that is, in a given patient or patient group. Therefore, personalized GEMs and their predictions of a patient's response to different drugs can be extremely useful for guiding precision medicine approaches.

## Concluding remarks

Here, we reviewed some of the publicly available human transcriptomics data resources with a focus on the expression data for protein‐coding genes. Tissue‐restricted and tissue‐enriched genes can be consistently defined in a genome‐wide manner by two independent datasets generated using either fresh surgically removed tissues or postmortem tissues taken within 24 hours after the death of the individual. Thus, comprehensive lists of protein‐coding genes can be compiled for all the major tissues of the human body (see Table EV1), with their quantitative expression profiles generated by deep sequencing of the transcriptome.

The use of high‐quality proteomics and transcriptomics data in combination with metabolic modeling allows for functional analyses in the context of different pathologies, for example, by comparing GEMs reconstructed using data from healthy and diseased subjects. On the one hand, a comparison of the healthy vs. diseased GEM topology can provide insights into how cancer metabolism differs from metabolism of the healthy tissue (Gatto *et al*, 2014; Björnson *et al*, 2015; Zhang *et al*, 2015). Furthermore, GEMs can be used for identifying drug targets (Agren *et al*, 2014), and therefore, their integration with omics data generated in a clinical setup can be applied to guide precision medicine in different disease types. Further improvement and expansion of GEMs to cover other biological processes, for example, protein secretion pathways and protein synthesis (Feizi *et al*, 2013), will allow this modeling framework to capture dysfunction of key cellular pathways in a range of different pathologies, potentially leading to the identification of new treatment strategies and biomarkers.

The transcriptomics data can be complemented with immunohistochemistry to define protein localization in the subcompartments of each tissue and organ down to the single‐cell level (www.proteinatlas.org). Moreover, extending these tissue profiles to include splice variants and protein modifications is important for improving our understanding of the role of the isoform proteome and post‐translational modifications in human physiology and disease. Finally, spatial proteomics using fluorescent‐based antibody profiling (Marx, 2015) can provide even higher resolution with precise localizations of the corresponding proteins down to subcellular compartments and various substructures. The integration of transcriptomics data with other large‐scale data, such as mass spectrometry‐based proteomics, antibody‐based profiling, and metabolomics, can thus generate an important molecular knowledge base for systems biology of human health and disease.

## Conflict of interest

The authors declare that they have no conflict of interest.

## Supporting information



Table EV1

Table EV2
