# Supplementary material for: Transcriptomics resources of human tissues and organs
Source: Mol Syst Biol. 2016 Apr 4;12(4):862. doi: 10.15252/msb.20155865 (PMC4848759; doi:10.15252/msb.20155865)
Supplement: Supplementary file 2 — Table EV2 [file MSB-12-862-s002.doc]

**Table EV2.** The overlapping and non-overlapping tissue-elevated genes, as shown in Figure 3A.

|  | **Both** | **Only HPA** | **Only GTEx** |
| --- | --- | --- | --- |
| Testis | 1136 | 76 | 108 |
| Brain | 393 | 32 | 48 |
| Liver | 189 | 4 | 5 |
| Skin | 106 | 6 | 88 |
| Skeletal muscle | 103 | 16 | 3 |
| Kidney | 75 | 6 | 0 |
| Esophagus | 59 | 5 | 3 |
| Pancreas | 45 | 0 | 5 |
| Fallopian tube | 42 | 19 | 0 |
| Adrenal | 37 | 9 | 5 |
| Heart | 34 | 1 | 0 |
| Stomach | 31 | 4 | 0 |
| Thyroid | 27 | 3 | 3 |
| Small intestine | 27 | 0 | 4 |
| Lung | 24 | 0 | 1 |
| Prostate | 22 | 4 | 1 |
| Spleen | 21 | 0 | 6 |
| Colon | 15 | 0 | 0 |
| Adipose tissue | 15 | 9 | 0 |
| Ovary | 9 | 0 | 0 |
